# TIMI Frame Count and Adverse Events in Women with No Obstructive Coronary Disease: A Pilot Study from the NHLBI-Sponsored Women's Ischemia Syndrome Evaluation (WISE)

**DOI:** 10.1371/journal.pone.0096630

**Published:** 2014-05-06

**Authors:** John W. Petersen, B. Delia Johnson, Kevin E. Kip, R. David Anderson, Eileen M. Handberg, Barry Sharaf, Puja K. Mehta, Sheryl F. Kelsey, C. Noel Bairey Merz, Carl J. Pepine

**Affiliations:** 1 Division of Cardiovascular Medicine, University of Florida, Gainesville, Florida, United States of America; 2 Graduate School of Public Health, University of Pittsburgh, Pittsburgh, Pennsylvania, United States of America; 3 College of Nursing, University of South Florida, Tampa, Florida, United States of America; 4 Division of Cardiology, Brown University, Providence, Rhode Island, United States of America; 5 Division of Cardiology, Barbra Streisand Women's Heart Center, Heart Institute, Cedars-Sinai Medical Center, Los Angeles, California, United States of America; Indiana University School of Medicine, United States of America

## Abstract

**Background:**

TIMI frame count (TFC) predicts outcomes in patients with obstructive coronary artery disease (CAD); it remains unclear whether TFC predicts outcomes in patients without obstructive CAD.

**Methods:**

TFC was determined in a sample of women with no obstructive CAD enrolled in the Women's Ischemia Syndrome Evaluation (WISE) study. Because TFC is known to be higher in the left anterior descending artery (LAD), TFC determined in the LAD was divided by 1.7 to provide a corrected TFC (cTFC).

**Results:**

A total of 298 women, with angiograms suitable for TFC analysis and long-term (6–10 year) follow up data, were included in this sub-study. Their age was 55±11 years, most were white (86%), half had a history of smoking, and half had a history of hypertension. Higher resting cTFC was associated with a higher rate of hospitalization for angina (34% in women with a cTFC >35, 15% in women with a cTFC ≤35, P<0.001). cTFC provided independent prediction of hospitalization for angina after adjusting for many baseline characteristics. In this cohort, resting cTFC was not predictive of major events (myocardial infarction, heart failure, stroke, or all-cause death), cardiovascular events, all-cause mortality, or cardiovascular mortality.

**Conclusions:**

In women with signs and symptoms of ischemia but no obstructive CAD, resting cTFC provides independent prediction of hospitalization for angina. Larger studies are required to determine if resting TFC is predictive of major events in patients without obstructive coronary artery disease.

## Introduction

Women with symptoms and signs of ischemia, referred for invasive coronary evaluation, often have no evidence of obstructive coronary artery disease (CAD) [1]. We and others have identified that symptomatic patients with non-obstructive CAD have an elevated risk of adverse outcomes and all-cause mortality compared with cohorts without symptoms and/or signs of ischemic heart disease [2], [3]. About 45% to 60% of such patients have coronary vascular dysregulation (endothelial or non-endothelial dependent macro- or microvascular dysfunction) capable of causing ischemia with invasive provocative testing [1], [4]. Additionally, we and others have linked coronary vascular dysregulation with adverse outcomes. Thus, additional indices of coronary vasomotor function, beyond standard anatomy from angiography, would be useful to improve risk stratification of these patients [5]–[9].

The Thrombolysis in Myocardial Infarction (TIMI) frame count (TFC) provides a simple angiographic index of coronary blood flow that does not require additional coronary artery instrumentation [10]. The intra- and inter-observer reproducibility of TFC is good, and dye injection rate and catheter size do not affect its measurement [11], [12]. The TFC has correlated with other invasive and non-invasive measures of coronary blood flow [13]–[16]. Further, TFC estimates of coronary flow after reperfusion for acute myocardial infarction predict short- and long-term clinical outcomes [17]–[19]. However, the prognostic implication of an abnormal TFC in patients without acute myocardial infarction or obstructive CAD and with suspected microvascular dysfunction remains unclear. Accordingly, we aimed to determine if TFC at rest is predictive of adverse outcomes in patients without obstructive CAD.

## Methods

### Patients

The Women's Ischemia Syndrome Evaluation (WISE) study (clinicaltrials.gov Identifier NCT00000554) is a National Heart, Lung and Blood Institute–sponsored study aimed at improving diagnostic evaluation and understanding of pathological mechanisms of ischemic heart disease in women. The WISE protocol was approved by the relevant institutional review boards (IRB) (University of Florida IRB, Allegany General Hospital IRB, University of Pittsburgh IRB, University of Alabama at Birmingham IRB), and has previously been described [20]. All participants provided written informed consent to participate in this study. The consent procedure and form were approved by the local IRBs listed. Briefly, women older than 18 years of age with symptoms and signs of ischemia undergoing clinically indicated angiograms were followed for clinical outcomes. Major exclusion criteria included comorbidities likely to compromise follow up. Initial evaluation in addition to coronary angiography included collection of demographics, medical history, symptom data, physical examination, and blood sampling for lipids, reproductive hormones, and inflammatory markers.

### Coronary Angiography and TIMI frame counts

**Figure 1:**
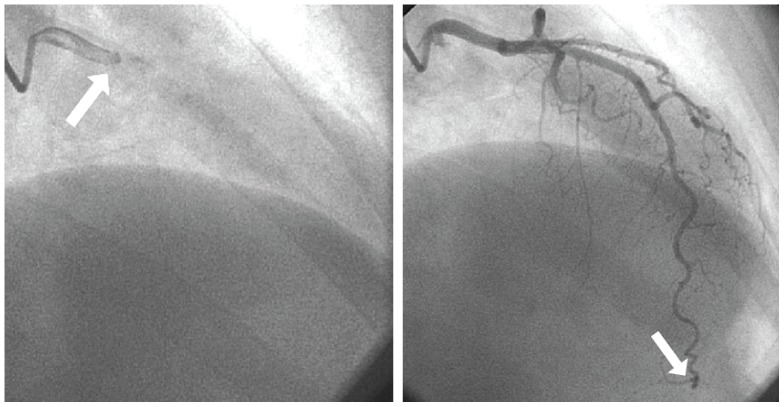
TIMI Frame Count. The first frame used to determine the TIMI Frame Count is the frame in which dye fully enters the artery of interest (left, arrow). The last frame that is counted is the frame when dye enters the distal landmark branch (right, arrow).

Coronary angiography was performed at the clinical sites according to standard methods. Qualitative and quantitative coronary angiographic analyses were conducted by a core laboratory masked to patient data [21]. As previously described, the TFC was determined as the number of cine frames required for contrast to reach standardized distal coronary landmarks [10]. The first frame used for TFC is the frame in which dye fully enters the artery of interest. The last frame that is counted is the frame when dye enters the distal landmark branch (Figure 1). The distal landmark branch of the left anterior descending (LAD) artery was the distal bifurcation. In normal coronaries, the TFC in the LAD is on average 1.7 times longer than the TFC in the other coronary arteries. Therefore, the LAD frame counts were divided by 1.7 to provide a corrected TFC (cTFC) [10]. Those patients who had an angiogram recorded in a way that allowed the determination of TFC by the core lab and were found not to have obstructive CAD are included in this analysis.

### Follow-up for adverse events

After the baseline angiogram and coronary reactivity testing, the WISE women had protocol-directed yearly follow-up. During telephone contact, a scripted interview was completed by an experienced nurse or physician at the respective center. Each patient or family member was queried for occurrence of major adverse cardiac events or hospitalizations. Telephone follow-up was terminated at a maximum of 8 years, with a median follow-up of 6 years. Additionally, after the phone follow-up period, a second phase of follow-up was performed with a search of the National Death Index for those patients still alive at last contact who had not withdrawn consent. This extended the follow-up for mortality to approximately 10 years. In the event of death, a death certificate and/or physician narrative was obtained. All deaths were adjudicated as cardiovascular (CV) or non-CV by a committee of senior WISE investigators blinded to angiographic findings. Women sustaining multiple events were counted only once and by the initial event.

### Statistical Analysis

Baseline characteristics are described by mean and standard deviation for continuous variables and percentages for categorical variables. The Kaplan-Meier method was used to estimate 10-year CV events rates by tertiles of cTFC, and compared by use of the log-rank statistic. To determine the cTFC cutpoint for predicting hospitalization for angina, we generated a receiver-operator characteristic (ROC) curve using logistic regression. The cTFC value corresponding to the point of the curve closest to 100% sensitivity and specificity was selected and verified in subsequent runs of incremental cut-points near that value. Multivariate Cox proportional hazards regression was used to examine the relationship between baseline characteristics and adverse outcomes. Baseline characteristics were chosen for entry into multivariable Cox models on the basis of their discrimination between those patients above and below the optimal cTFC cutpoint, as well as on univariate associations with adverse outcomes of P<0.20. A combination of forward and backward selection procedures were used to aid in determining the best model of independent predictors. This was followed by forcing potential confounders into the models and determining their effect on the relationship of interest. The likelihood ratio test was used to compare the incremental goodness of fit of nested models. All tests were 2-sided, and P≤0.05 was considered statistically significant. All analyses were performed with SAS software version 9.3 (SAS Institute). *Sample Size Considerations*. Using previously published data for women in the WISE with non-obstructive coronary disease [9], we assumed a reference 6-year event-free survival rate of 91% for such women without coronary microvascular dysfunction (e.g. normal cTFC). Event-free survival was taken as freedom from first occurrence of death (all-cause), nonfatal MI, non-fatal stroke, or hospitalization for heart failure. We assumed that women with abnormal coronary microvascular function (e.g. high cTFC) would have reduced event-free survival. This was expressed as a hazard ratio of 2.5 (corresponding to a 6-year event free survival rate of approximately 79%) with proportional hazard assumption. Assuming 5% censoring, we would need 300 women to achieve 80% power and need 500 women to achieve 95% power, using a two-sided log-rank test with alpha level of 0.05. SAS procedure PROC POWER was used to perform the power analysis. We suspected we were underpowered in our comparison of the incidence of the binary outcome of major events between TFC groups, and we used the Kaplan-Meier estimated rates of major events between our two TFC groups in this pilot study (20% vs. 13%) to determine the sample size necessary to detect a statistically significant difference at a power of 80% and an alpha of 0.05 using the SAS procedure PROC POWER with two-sided Pearson's chi square test.

## Results

### Baseline Characteristics

We report on a subgroup of 298 women without obstructive CAD (no stenosis ≥50% diameter reduction), whose angiograms could be retrieved and were suitable for analysis by TFC, and for whom clinical outcome data were available. Their baseline demographics are shown in Table 1. Their mean age was 55±11 years, most were white (86%), nearly half had a history of smoking, and half had a history of hypertension.

**Table 1:** Baseline characteristics and risk factors.

Characteristic		All women (n = 298)	cTFC ≤35 n = 179	cTFC >35 n = 119	P (unad-justed)	P (age-adjusted)
Demographics						
	Age (y)	55±11	56±11	54±10	0.20	-
	Postmenopausal (%)**	210/296 (71)	72	69	0.53	-
	White race (%)	257 (86)	90	80	0.009	0.013
	HS educ. or more (%)	243/294 (83)	82	83	0.97	0.94
Body size						
	Waist circumference (in)	36.8±6.8	36.0±5.5	38.2±8.5	0.027	0.020
	BMI	30.3±7.1	29.8±6.6	31.0±7.8	0.17	0.20
	Waist/hip ratio	0.85±0.12	0.85±0.11	0.87±0.14	0.20	0.18
Self-reported risk factors						
	Hx diabetes (%)	42/297 (14)	11	19	0.07	0.053
	Family hx of CAD (%)	181/290 (62)	59	67	0.16	0.18
	Hx hypertension (%)	143/296 (48)	47	51	0.48	0.34
	Hx dyslipidemia (%)	116/275 (42)	43	40	0.65	0.74
	Ever smoker (%)	151/297 (51)	54	47	0.24	0.20
	Current smoker (%)	58/297 (20)	20	19	0.75	0.50
	High stress (%)	117 (39)	40	39	0.86	0.70
Hemodynamic measures						
	Systolic BP (mmHg)	133±20	134±20	131±19	0.21	0.33
	Diastolic BP (mmHg)	76±10	77±11	76±10	0.37	0.34
	Pulse pressure	57±16	58±16	56±16	0.25	0.44
	Mean arterial pressure	95±12	96±12	94±11	0.22	0.28
Lab values						
	LDL-C (mg/dl)	111±37	112±39	109±33	0.52	0.58
	HDL (mg/dl)	53±13	53±13	53±13	0.99	0.82
	Triglycerides (mg/dl) (medians[IQR])*	113 [72, 169]	122 [76, 169]	96 [70, 172]	0.37	0.55*
	Total cholesterol	191±43	192±44	191±41	0.92	0.94
	Fasting blood glucose	104±39	103±38	107±40	0.33	0.31
	Creatinine (mg/dl)	0.79±0.18	0.80±0.19	0.78±0.16	0.48	0.61
	Hemoglobin (g/dl)	13.0±1.4	13.1±1.4	12.9±1.3	0.25	0.21
Medication use						
	Any antihypertensive use (%)	167/283 (59)	60	57	0.62	0.74
	Any lipid lowering meds (%)	52/297 (18)	19	15	0.41	0.45
	Ever HRT use (%)	160/293 (55)	56	52	0.42	0.55
	Current HRT use (%)	116/294 (39)	40	38	0.70	0.83
	Statins (%)	39/297 (13)	13	14	0.86	0.77
	ACE inhibitors (%)	51/269 (19)	18	20	0.66	0.51
	Beta blockers (%)	89/297 (30)	29	31	0.67	0.65
	Aspirin (%)	138/296 (47)	49	42	0.23	0.27
	Diuretics (%)	63/297 (21)	19	24	0.25	0.19
Other variables						
	CAD severity score (medians[IQR])*	5.0 [5.0, 7.5]	5.0 [5.0, 7.5]	5.0 [5.0, 6.2]	0.57	0.72*
	Functional capacity (DASI)	21.4±14.9	22.6±15.3	19.7±14.2	0.11	0.10
	Unstable angina past 6 wks	74/266 (28)	27	29	0.72	0.81

cTFC  =  corrected TIMI frame count; HS educ  =  High School education; BMI  =  body mass index; Hx  =  history; HRT  =  hormone replacement therapy; CAD  =  coronary artery disease.

*Because of skewed distribution, medians [interquartile ranges] are given and Log transformations were used to estimate the age-adjusted p-values.

**For frequencies, where there were missing values, the statistic given is: no. with the condition/no. available (%).

### Angiography and TFC

These women, in aggregate, had normal overall left ventricular systolic function with a mean ejection fraction of 66±10%. The mean cTFC was 33.9±10.0 with a range of 18 to 91.

### Adverse Outcomes

Adverse event rates were compared between patients in the various cTFC tertiles (cTFC ≤28, 29–37, and >37). During a median follow up of 6 years, the Kaplan-Meier event rate for angina hospitalization was highest for those women in the highest cTFC tertile (35%, vs. 24% in the middle cTFC tertile, and 19% in the lowest cTFC tertile, P = 0.022). However, the rates of major events (myocardial infarction, heart failure, stroke, or all-cause death), CV events (myocardial infarction, heart failure, stroke, or CV death), all-cause mortality, or CV mortality were not statistically different between women in the various cTFC tertiles.

**Figure 2:**
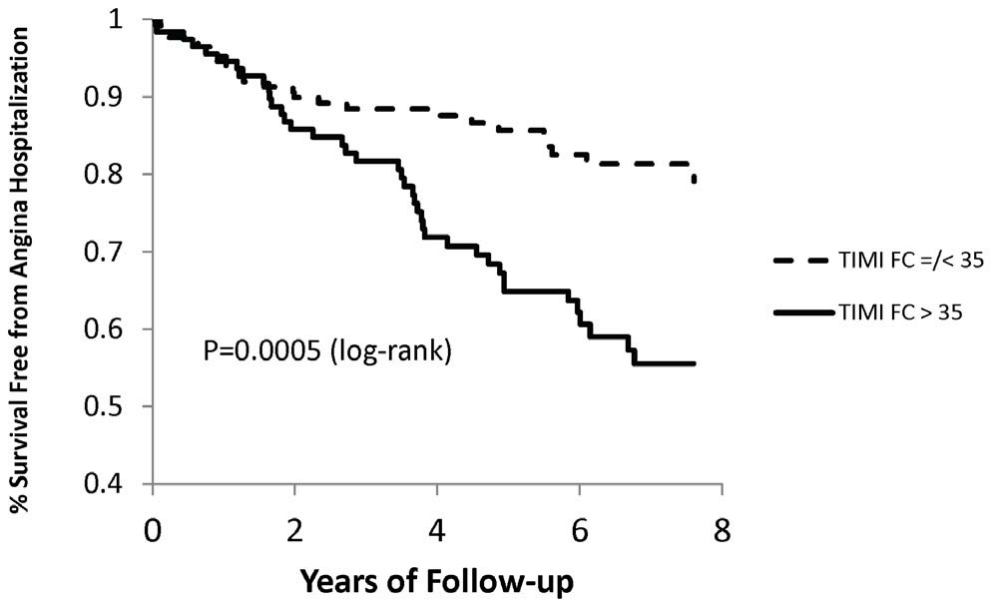
Survival free of hospitalization for angina according to TIMI Frame Count groups. Kaplan-Meier analysis of survival free of hospitalization for angina.

By ROC analysis, a cTFC of >35 provided the best cut-point for predicting hospitalization for angina, with a sensitivity of 61% and a specificity of 66%. The area under the curve was 0.63 (95% confidence interval [CI] 0.57 to 0.7). Adverse event rates in those patients above versus at or below a cTFC of 35 are shown in Table 2. Thirty four percent of women with a cTFC >35 were hospitalized for angina, whereas only 15% of women with a cTFC ≤35 were hospitalized for angina (P<0.001) (Figure 2).

**Table 2:** Adverse Event Rates between TIMI Frame Count Groups.

Event*	All Women (n = 298)		cTFC ≤35 n = 179		cTFC >35 n = 119		P (unadjusted)
	# Events	K-M Rate**	# Events	K-M Rate**	# Events	K-M Rate**	
Major event	38	16%	19	13%	19	20%	0.30
CV event	33	13%	18	12%	15	15%	0.70
All-cause mortality	24	11%	12	10%	12	12%	0.55
CV mortality	13	6%	7	5%	6	7%	0.88
Angina hospitalization	60	26%	24	17.5%	35	38%	0.0005
6-year CV event or angina hospitalization	73	26%	31	19%	42	37%	0.001

cTFC  =  corrected TIMI Frame Count; K-M  =  Kaplan-Meier; Major event  =  MI, heart failure, stroke, or all-cause death; CV (cardiovascular) Event  =  MI, heart failure, stroke, or CV death.

*Mortality is estimated for up to 10 years of follow up. Non-fatal events are estimated for up to 6 years of follow up.

**Note that the K-M estimated rate is not equivalent to the number of events per n of women.

Baseline characteristics were examined for their prediction of hospitalization for angina. Patients with white race had a lower risk of hospitalization for angina (age-adjusted HR = 0.43 [95% CI 0.24, 0.76], P = 0.004), whereas a history of unstable angina in the preceding 6 weeks predicted hospitalization for angina (age-adjusted HR 1.79 [95% CI 1.05, 3.03], P = 0.03). Further, the use of various medications at baseline was predictive of angina hospitalization: anti-hypertensives (age-adjusted HR 1.96 [95% CI 1.15, 3.35], P = 0.014); ACE inhibitors (age-adjusted HR 2.17 [95% CI 1.22, 3.86], P = 0.009); and diuretics (age-adjusted HR 2.09 [95% CI 1.2, 3.62], P = 0.009).

When added to a predictive model that included cTFC at cut-point 35, a history of unstable angina and diuretic use were not independently associated with hospitalization for angina. In contrast, when white race was included in a model that contained cTFC at cut-point 35, it did provide an independent predictor of lower risk of hospitalization for angina (HR 0.53 [95% CI 0.3, 0.94], P = 0.03). Additionally, when ACE inhibitor use was included in a model that contained cTFC at cut-point 35, it was associated with a higher risk of hospitalization for angina (HR 2.04 [95% CI 1.15, 3.59], P = 0.014). However, when ACE inhibitor use, white race, and cTFC at cut-point 35 were all included in a predictive model of hospitalization for angina, only cTFC provided independent prediction of hospitalization for angina.

## Discussion

Microvascular coronary dysfunction is prevalent in patients presenting with chest pain who have no evidence of obstructive CAD on standard angiography, and is associated with adverse outcomes. Microvascular coronary dysfunction is commonly diagnosed by evaluating coronary blood flow before and after administration of adenosine with a Doppler-tipped guide wire in the coronary. TFC does not require placement of a wire in the coronary artery, and has been shown to correlate with coronary blood flow and prognosis in patients with acute coronary events. The correlation of TFC with adverse outcomes in patients with no obstructive disease but suspected microvascular coronary dysfunction is less clear.

In the 298 women included in this study who had chest pain but no evidence of obstructive CAD, a high cTFC was associated with an increased risk of hospitalization for angina. Moreover, a high cTFC remained the strongest predictor of hospitalization for angina after controlling for baseline characteristics. Interestingly, ACE inhibitor use was also associated with an increased risk of angina hospitalization. We have previously shown that patients randomized to an ACE inhibitor had greater improvement in angina severity as compared to those who received placebo [22]. So these data are biased by WISE investigators likely prescribing an ACE-I for those with more severe angina. Additionally, follow-up blood pressure for our current cohort was higher than the baseline blood pressure in those patients randomized to an ACE inhibitor in the Pauly et al trial [22]. We suspect that baseline ACE inhibitor use in our current cohort is likely a marker of more advanced vascular disease, which may have contributed to an increased risk of angina hospitalization. In this pilot investigation, cTFC was not associated with a statistically significant increase in risk of major events (myocardial infarction, heart failure, stroke, or all-cause death), CV events, all-cause mortality, or CV mortality.

Studies comparing TFC to more invasive measures of coronary vasomotion (endothelial or non-endothelial dependent macro- or microvascular function) have provided various results. Many studies have shown that TFC does correlate with measures of coronary vasomotion [23]–[26], whereas others have shown no correlation of TFC and coronary flow reserve [27]. Most of these studies were small. Some of these studies included patients with obstructive CAD and others excluded patients with obstructive CAD. Some of these studies determined the change in TFC from baseline to the TFC at hyperemia, and correlated this change in TFC with measures of coronary vasomotion, whereas some looked at only the correlation of TFC at rest with measures of coronary vasomotion. Therefore, it remains unclear if TFC at rest can replace evaluation of coronary vasomotion with more traditional methods. Because TFC at rest does not require instrumentation of the coronary artery nor vasoactive medication, future study evaluating the correlation of TFC at rest with CFR determined after administration of adenosine and change in coronary artery diameter and coronary blood flow after acetylcholine in patients without obstructive CAD remains warranted.

The major limitation of our study is the relatively small number of major events. Because of the small number of events, the current analysis provides low statistical power to find a relationship between TFC and major events. In order to have appropriate power to determine if there is a significant difference in major event rates between TFC groups, we estimate that we would need approximately 880 patients.

In conclusion, in women with signs and symptoms of ischemia but no obstructive CAD, resting cTFC provides independent prediction of hospitalization for angina. Larger studies are required to determine if resting cTFC is predictive of major events in patients without obstructive coronary artery disease.
